# The effectiveness of educational methods for cricothyroid membrane identification by dental students: A prospective study using neck photographs and tracheotomy trainers

**DOI:** 10.1002/cre2.167

**Published:** 2019-03-04

**Authors:** Takashi Goto, Toshiyuki Kishimoto, Satoru Sakurai

**Affiliations:** ^1^ Department of Dental Anesthesiology, Division of Oral Pathogenesis and Disease Control Asahi University School of Dentistry Mizuho City Japan

**Keywords:** cricothyroid membrane, cricothyrotomy, dental students, emergency, practicums

## Abstract

The purpose of this study was to evaluate the accurate identification of the cricothyroid membrane (CTM) by fifth grade dental students, before undergoing the relevant anesthesiology practicum. Moreover, we aimed to determine the educational effectiveness of the cricothyrotomy practicum in anesthesiology. Before the lecture and without prior notification, 119 students were provided with a photograph of a man's neck and instructed to attach a blue sticker to the cricothyrotomy puncture site and to a palpable tracheotomy trainer, after applying sufficient palpation. After this, students attended a 60‐min lecture on the emergency airway management method. Two and 16 days after the lecture and practicum, students were presented with a new neck photograph and the tracheotomy trainer and asked again to place stickers (red stickers: at 2 days; green stickers: at 16 days) on the cricothyrotomy penetration site. The data were analyzed with an image processing software, by superimposing the 119 stickers on the neck photographs and tracheotomy trainers, to visually examine the accuracy of CTM identification. The rate of correct sticker placement in the neck photographs was 41.2% before the lecture, 80.7% 2 days after the lecture, and 77.3% 16 days after the lecture (before vs. 2 and 16 days after, *p* value < 0.01). For the tracheotomy trainer, the rate was 36.1% before the lecture, 97.5% 2 days after the lecture, and 94.1% 16 days after the lecture (before vs. 2 and 16 days after, *p* value < 0.01). Furthermore, the proportion of students with mistakes above and below the CTM was higher than that of students with mistakes to the right or left. In conclusion, the rate of accurate CTM identification among dental students was low before they underwent the relevant practicum, but most students were able to identify the CTM accurately after the lecture and practicum in a small class.

## 1 INTRODUCTION

Dental treatment includes airway structures in the operative field, and airway complications during a dental procedure can result in patient mortality. Of the 33 dental treatment‐related deaths in Japan during 2002–2012, eight (24%) were caused by airway obstructions (Sato et al., 2014; Sato, Katsumura, & Kobatashi, 2013). In particular, fatal accidents often occur due to airway edema caused by anaphylaxis, airway obstruction due to the entry of surgical instruments and extracted teeth into the oral cavity, and intraoperative and postoperative bleeding (Sato et al., 2013; Sato et al., 2014).

According to the airway management guidelines for the introduction of general anesthesia, published by the American Society of Anesthesiologists (Japanese Society of Anesthesiologists, 2014) and Japanese Society of Anesthesiologists (Apfelbaum et al., 2013), cricothyrotomy is recommended for airway management in severe emergency circumstances, such as airway obstruction. The cricothyroid membrane (CTM) is suitable for emergency penetration due to the following reasons: (a) it can be identified easily, as it is located close to the surface of the neck; (b) it contains few blood vessels; (c) the thyroid is not affected by CTM penetration; and (d) its posterior wall is difficult to penetrate (Dover, Howdieshell, & Colborn, 1996). However, it is difficult to accurately identify and penetrate the CTM in emergency situations. In particular, it is extremely difficult to identify the CTM with palpation in female patients (Aslani et al., 2012; Campbell et al., 2014; Hiller, Karni, Cai, Holcomb, & Hagberg, 2016; Lamb et al., 2015) and patients with obesity (Campbell et al., 2014; Lamb et al., 2015; McGill, Clinton, & Ruiz, 1982). Therefore, accurate identification of the CTM is the key for successful cricothyrotomy.

Dental students at Asahi University School of Dentistry complete courses in anatomy (lectures and practicum with cadavers) and anesthesiology (lectures) by the fourth grade. In the fifth grade, they perform basic and clinical practicums of airway management procedures in the anesthesiology class, such as mask ventilation, use of supraglottic airway, tracheal intubation, and cricothyrotomy, using models and patients. Therefore, the primary objective of this study was to evaluate whether dental students in the fifth grade could identify the CTM accurately, before undergoing the relevant anesthesiology practicum. The second objective was to examine the educational effectiveness of the cricothyrotomy practicum in anesthesiology.

## 2 METHODS

Ethics approval was granted by Asahi University, Japan (approval number 25154). Data for this study were collected from May 2014 to February 2015. The subjects included 119 dental students in the fifth grade, all of whom provided informed consent. The students attended anatomy and anesthesiology lectures and completed the anatomy practicum (a college course designed to give a student supervised practical knowledge of a subject previously studied theoretically), using cadavers by the fourth grade. The Japanese dental education system involves a 6‐year program.

**Figure 1:**
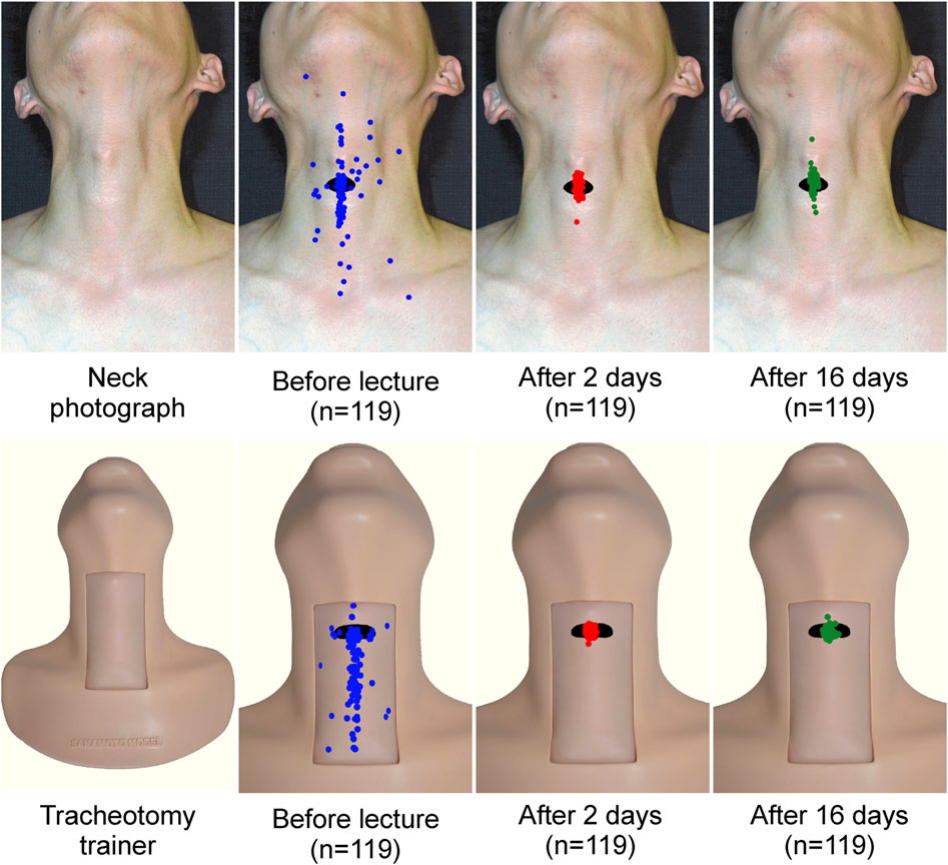
Penetration sites marked on the neck photograph and tracheotomy trainer before, 2 days after, and 16 days after the lecture. The neck photograph and tracheotomy trainer presented to the students are shown, respectively, in the upper and lower panels. Blue, red, and green dots show the penetration sites before, 2 days after, and 16 days after the lecture. The black circle indicates the actual position of the cricothyroid membrane

Before the lecture, without prior notification, we presented the students with a photograph of the neck of a man and instructed them to attach a blue sticker to the cricothyrotomy puncture site (Figure 1). After this, the students were presented with a palpable tracheotomy trainer (Sakamoto Tracheotomy Trainer; Sakamoto Model Co., Osaka, Japan), and after applying sufficient palpation, students were asked to attach another blue sticker to the cricothyrotomy puncture site (Figure 1). The neck photograph and tracheotomy trainer were adopted to emphasize visual inspection and palpation, respectively. The size of the sticker (5 mm diameter) was similar to the size of the cannula tip from Quicktrach (VBM Medizintechnik GmbH, Sulz am Neckar, Germany), a cricothyrotomy kit. The sticker application was performed in an examination format, each student performing the entire procedure independently. Following this, students attended a 60‐min lecture on emergency airway management methods covering topics such as neck anatomy, mask ventilation, use of supraglottic airway, tracheal intubation, and cricothyrotomy. In addition, students were given the opportunity to palpate one another's CTM. After the lecture, the students underwent a practicum for tracheal intubation using the tracheal intubation trainer and for cricothyrotomy using the tracheotomy trainer and Quicktrach. Each student practiced the procedure twice. The lecture and practicum were conducted in a small class of eight to nine students. We emphasized that the penetration site should not deviate from the midline. Two and 16 days after the lecture and practicum, a new neck photograph and the tracheotomy trainer were presented, and the students were instructed to place stickers (red stickers: 2 days after; green stickers: 16 days after) on the cricothyrotomy penetration site. The time of the measurements (2 and 16 days after the practicum) was established on the basis of the curriculum schedules of the university. The neck photographs, on which the stickers were placed, were digitized using a scanner. Although the tracheotomy trainers, on which the stickers were placed, were digitized using standardized photographs taken with an imaging instrument (Copy Stand Column CSC‐20; LPL Co., Ltd. Saitama, Japan). These electronic data were analyzed with the image processing software Adobe^®^ Photoshop^®^ Elements 11 (Ver. 11.0; Adobe System Incorporated, USA), by superimposing the 119 stickers on the neck photographs and tracheotomy trainers, to visually examine the accuracy of the penetration sites.

**Figure 2:**
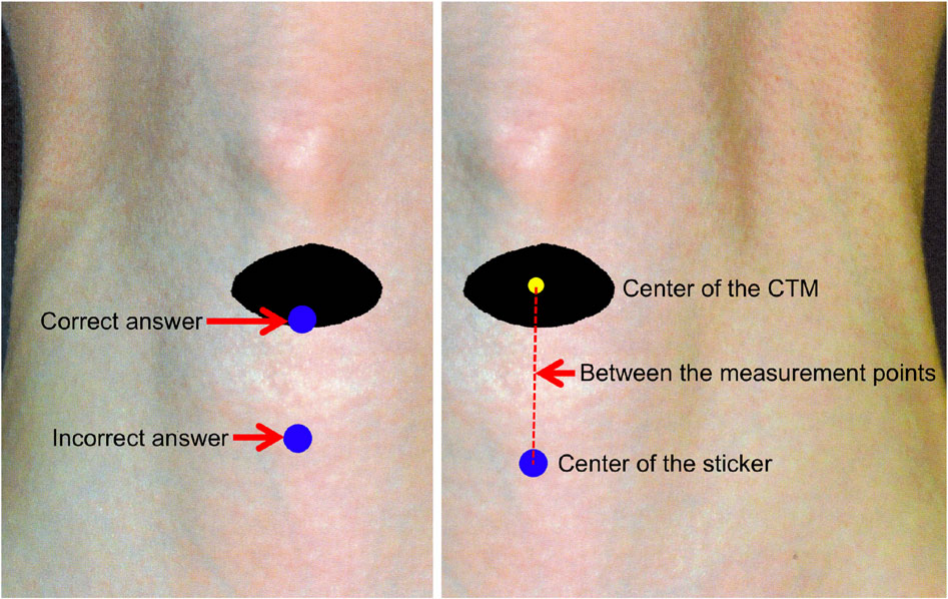
Measurement method (the distance from the correct answer to the center of the placed sticker). Left panel: the difference between the correct and incorrect answers. The answer was correct when the placed sticker was inside the cricothyroid membrane (CTM; black margin). Right panel: the distance between the measurement points, that is, the center of the cricothyroid membrane and the center of the placed sticker, was measured (mm)

The parameters evaluated were as follows: (a) the rate of correct sticker placement; (b) the distance from the center of the CTM to the sticker position on the neck photograph or tracheotomy trainer; (c) the area of the sticker placement; (d) the percentage of participants who placed the stickers ≥5.0 mm mediolateral to the CTM position; and (e) the percentage of participants who placed the stickers above or below the CTM position. We examined the accuracy of CTM identification on the basis of these measures. The rate of correct sticker placement was analyzed by determining the percentage of cases in which the center of the sticker was at the CTM site on the neck photograph or tracheotomy trainer (Figure 2). To measure the distance from the center of the CTM to the center of the sticker, we utilized the use of a measurement point as shown in Figure 2. To determine the area of the sticker, we measured the sticker on the superimposed image using ImageJ 1.51 s (National Institutes of Health, Bethesda, MD); the area measurements were expressed in pixels. The percentage of participants who placed the stickers ≥5.0 mm from the CTM site was determined using the center line connecting the thyroid cartilage and jugular notch. The cutoff value of 5.0 mm was determined on the basis of previous studies (Aslani et al., 2012; Hiller et al., 2016; Lamb et al., 2015).

## 3 STATISTICAL ANALYSIS

Statistical analyses were performed using SPSS (IBM^®^ SPSS^®^ Statistics, Version 23.0, for Linux x86‐64, Microsoft Windows, NY). Data were presented as numerical and mean ± *SD* (standard deviation [*SD*]) values. The distance from the center of the CTM to the center of the sticker on the neck photograph or tracheotomy trainer was measured before, 2 days after, and 16 days after the lecture, values were compared using repeated measures analysis of variance (ANOVA). Multiple comparison testing was performed using the Student–Newman–Keuls test, where *p* < 0.01 was considered to indicate statistical significance. Intergroup differences were examined with the paired *t* test, where *p* < 0.01 was considered to indicate statistical significance. The rate of correct sticker placement, the percentage of participants who placed the stickers ≥5.0 mm mediolateral to the CTM position, and the percentage of participants who placed the stickers above or below the CTM position were compared using the χ^2^ test, where *p* < 0.01 was considered to indicate statistical significance.

## 4 RESULTS

The image with 119 stickers superimposed on the neck photograph and tracheotomy trainer is shown in Figure 1. The ratios of students who identified the penetration site correctly, to those who placed the stickers outside the penetration site, are shown in Table 1.

**Table 1:** The percentages of participants (*n* = 119) who placed the stickers at the correct site and those who placed them outside the site (before, 2 days after, and 16 days after the lecture)

	Before the lecture *n* (%)	2 days after the lecture *n* (%)	16 days after the lecture *n* (%)	*p* value
Students who placed the stickers at the correct site				
Neck photograph	49 (41.2)	96 (80.7)	92 (77.3)	<0.01
Tracheotomy trainer	43 (36.1)	116 (97.5)	112 (94.1)	<0.01
Students who placed the stickers in the mediolateral direction				
Neck photograph	25 (21.0)	1 (0.8)	1 (0.8)	<0.01
Tracheotomy trainer	20 (16.8)	0 (0)	0 (0)	<0.01
Students who placed the stickers above or below the CTM				
Neck photograph	37 (31.1)	1 (0.8)	3 (2.5)	<0.01
Tracheotomy trainer	59 (49.6)	1 (0.8)	1 (0.8)	<0.01

*Note*. *n* (%) indicates the number of students and percentage (%). CTM: cricothyroid membrane.

### 4.1 Accuracy rate of correct sticker placement

The accuracy rate of correct sticker placement, in the neck photographs, before the lecture was 41.2%, 2 days after the lecture was 80.7%, and 16 days after the lecture was 77.3%. The ANOVA revealed that the rates obtained 2 and 16 days after the lecture were significantly greater than those obtained before the lecture (*p* < 0.01). On the tracheotomy trainer, the accuracy rate before the lecture was 36.1%, 2 days after the lecture was 97.5%, and 16 days after the lecture was 94.1%. Moreover, accuracy rates 2 and 16 days after the lecture were significantly greater than that before the lecture (*p* < 0.01; Table 1).

### 4.2 Distance from the center of the CTM to the center of the sticker on the photograph or tracheotomy trainer

**Figure 3:**
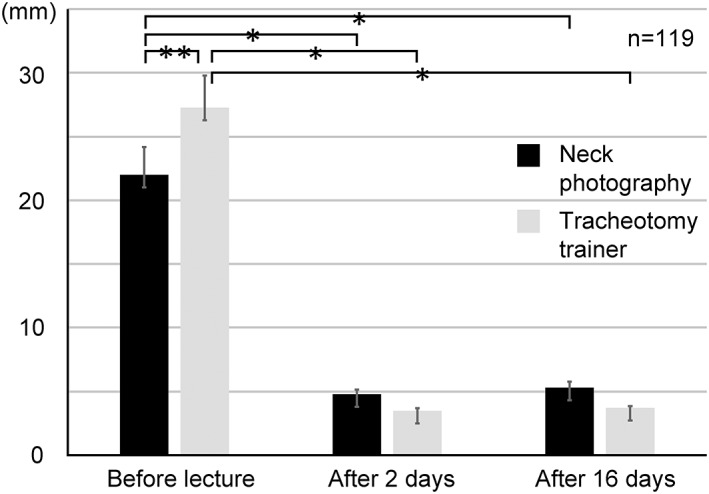
Distance between measurement points; *p < 0.01 and **p < 0.05

The distance from the center of the CTM to the center of the sticker, on the neck photographs, before the lecture was 22.0 ± 2.2 mm, 2 days after the lecture was 4.8 ± 0.4 mm, and 16 days after the lecture was 15.3 ± 0.5 mm. The ANOVA revealed that the distances measured 2 and 16 days after the lecture were significantly shorter than that measured before the lecture (*p* < 0.01). Conversely, on the tracheotomy trainer, the distance before the lecture was 27.3 ± 2.5 mm, 2 days after the lecture was 3.5 ± 0.2 mm, and 16 days after the lecture was 3.7 ± 0.2 mm. The ANOVA revealed that the distances measured 2 and 16 days after the lecture were significantly shorter than those measured before the lecture (*p* < 0.01). On comparison between distances on neck photographs and tracheotomy trainers, only the prelecture values for the neck photographs were shorter than that with the tracheotomy trainer (*p* < 0.05; Figure 3).

### 4.3 Area of the stickers

**Figure 4:**
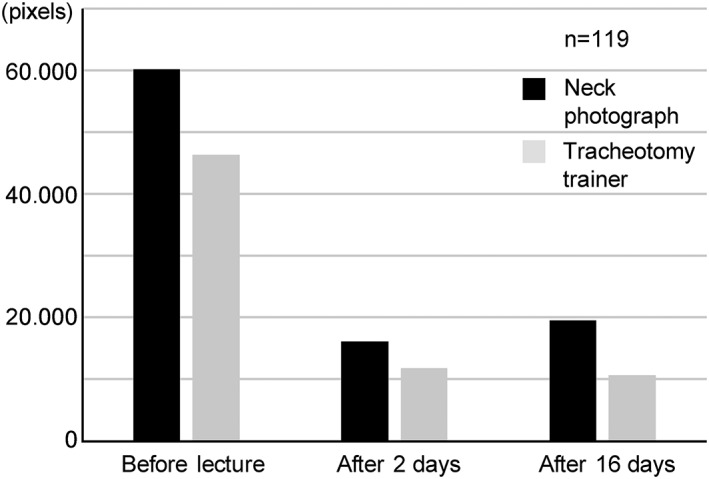
Area of the affixed stickers. The area of the stickers of the neck photograph was higher than that of the stickers of the tracheotomy trainer, but there was no statistically significant difference

The area of the stickers on the neck photograph, before the lecture was 601,565 pixels, 2 days after the lecture was 601,565 pixels, and 16 days after the lecture was 195,279 pixels. Whereas on the tracheotomy trainer, the area before the lecture was 463,261 pixels, 2 days after the lecture was 118,384 pixels, and 16 days after the lecture was 106,387 pixels (Figure 4).

### 4.4 Percentage of participants who placed the stickers ≥5.0 mm mediolateral to the CTM position

On the neck photographs, the percentage of participants who placed the stickers ≥5.0 mm mediolateral to the CTM position before the lecture was 21.0%, 2 days after the lecture was 0.8%, and 16 days after the lecture was 0.8%. The percentages obtained 2 and 16 days after the lecture were significantly smaller than those obtained before the lecture (*p* < 0.01). On the tracheotomy trainer, the percentage of participants placing the sticker mediolateral to the CTM before the lecture was 16.8%, whereas for both 2 and 16 days after the lecture was 0%. The percentages obtained 2 and 16 days after the lecture were significantly shorter than those obtained before the lecture (*p* < 0.01; Table 1).

### 4.5 Percentage of participants who placed the stickers above or below the CTM position

On the neck photographs, the percentage of participants who placed the stickers above or below the CTM position before the lecture was 31.1%, 2 days after the lecture was 0.8%, and 16 days after the lecture was 2.5%. The values obtained 2 and 16 days after the lecture were significantly smaller than those obtained before the lecture (*p* < 0.01). On the tracheotomy trainer, the percentage of participants misidentifying the CTM before the lecture was 49.6%, yet 2 and 16 days after the lecture were both 0.8%. The values obtained 2 and 16 days after the lecture were significantly smaller than those obtained before the lecture (*p* < 0.01; Table 1).

## 5 DISCUSSION

The purpose of this study was to evaluate the accurate identification of the CTM by fifth grade dental students, before undergoing the relevant anesthesiology practicum. Moreover, we aimed to determine the educational effectiveness of the cricothyrotomy practicum in anesthesiology. The rate of accurate CTM identification among dental students was low before they underwent the relevant practicum, but most students were able to identify the CTM accurately after the lecture and practicum in a small class.

Oxygenation by cricothyrotomy in patients in the “*cannot intubate*, *cannot ventilate* (*CICV*)” condition dramatically improves the symptoms, whereas incorrect penetration can lead to serious complications that may result in patient mortality (McGill et al., 1982). Various penetration kits are available for cricothyrotomy, but the basic procedure for cricothyrotomy involves a four‐step technique (Brofeldt, Panacek, & Richards, 1996). The first step is the basic procedure of “identification of the CTM,” which is the most important skill required for this technique.

We used a neck photograph of a male subject for identification of the CTM as visual identification of the thyroid and cricoid cartilages in photographs of female subjects is difficult, and previous studies have reported that identification of the CTM by palpation in female patients is difficult (Aslani et al., 2012; Hiller et al., 2016; Lamb et al., 2015). Lamb et al. (2015) reported that 72% of anesthesiology residents identified the CTM correctly in nonobese males; however, the correct answer rate among the dental students in our study was as low as 41.2% and 36.1% for the neck photograph and tracheotomy trainer, respectively (Table 1), suggesting that cricothyrotomy may be difficult for dentists. Nevertheless, when the CTM identification was reassessed after the lecture on cricothyrotomy in a small class of eight to nine students, the correct identification rate 2 and 16 days after, increased to 80.7% and 77.3%, respectively, for the neck photographs and 97.5% and 94.1%, respectively, for the tracheotomy trainer (Table 1). Furthermore, after the lecture, the distance from the center of the CTM site to the center of the stickers reduced for both the neck photograph and tracheotomy trainer (Figure 3), and the area of the sticker also reduced (Figure 4).

Surprisingly, the cricothyrotomy penetration sites, selected by many students before the lecture, deviated in the mediolateral direction (neck photograph: 21%; tracheotomy trainer: 16.8%; Table 1). Furthermore, numerous students selected penetration sites above or below the CTM before the lecture (neck photograph: 31.1%; tracheotomy trainer: 49.6%; Table 1). The prevalence of complications associated with cricothyrotomy has been reported to be 9–40% (McGill et al., 1982). In particular, deviation of the penetration sites in the mediolateral direction can increase the risk of serious complications as important blood vessels and nerves surround the CTM. Thus, during the lecture and practicum, after the first sticker placement, we emphasized that the penetration site should not deviate from the midline. Consequently, none of the students placed stickers outside the midline 2 and 16 days after the lecture. The number of students who placed the sticker above or below the penetration site in the tracheotomy trainer was around 50% before the lecture. This percentage is greater than the corresponding value for the neck photograph, likely due to students being unable to distinguish the texture of the thyroid cartilage, cricoid cartilage, and tracheal ring with palpation. It may be possible that the unevenness of the texture due to palpation complicated the comprehension of the anatomical structures. This finding indicates the importance of teaching the positional relationship of the anatomical structures in the midline, such as the thyroid cartilage, cricoid cartilage, and tracheal ring, as well as the palpation technique and instructions to avoid deviation from the midline. Therefore, these results show that dental students can gain proficiency in identification of the CTM, in a small class, where each procedure is carefully taught step by step in the lecture and practicum. This method is preferred to lectures in large classes (of 130 students) until the fourth grade.

The use of ultrasound devices has been reported to improve identification of the CTM (Siddiqui, Arzola, Friedman, Guerina, & You‐Ten, 2015), although only a few dental institutions, including university hospitals, are equipped with ultrasound devices, rendering such identification procedures unrealistic. Therefore, palpation is the primary method of identification among dentists. The palpation techniques used for CTM identification includes the following; the general palpation technique (i.e., top–down or bottom–up), four‐finger technique, and neck crease technique (Bair & Chima, 2015). It will therefore be necessary to determine the technique most suitable for dentists. One study reported that CTM identification rates with the four‐finger technique, neck crease technique, and general palpation technique were 46%, 50%, and 60%, respectively (Bair & Chima, 2015), suggesting that the general palpation technique can be recommended. However, the British guidelines for the management of unanticipated difficult intubation in adults, recommended the laryngeal handshake method during palpation, to identify the CTM (Frerk et al., 2015). The method of the laryngeal handshake method is detailed below (Sato et al., 2013). The index finger and thumb is used to grasp the top of the larynx (the greater cornu of the hyoid bone) and to roll it from side to side. The bony and cartilaginous cage of the larynx is a cone, which connects to the trachea (Sato et al., 2014). The fingers and thumb are slid down over the thyroid laminae (Japanese Society of Anesthesiologists, 2014). The middle finger and thumb are made to rest on the cricoid cartilage, and the index finger is used for palpating the CTM (Frerk et al., 2015). Moreover, the laryngeal handshake method has been shown to result in a higher CTM identification rate (62%) than the conventional method (33%; Drew & AcCaul, 2018). For this reason, it seems better to teach students this method in future.

In the various hospitals and departments, the incidence of emergent percutaneous intubation has been reported to be between 0.3% (Stephens, Kahntroff, & Dutton, 2009) and 0.8% (Walls, Brown, Bair, & Pallin, 2011). Conversely, its incidence in dental clinics is anticipated to be 0%, with few dentists practicing cricothyrotomy on a regular basis. However, as fatal accidents due to airway obstruction during dental treatment have previously been reported (Sato et al., 2013; Sato et al., 2014), determination of the educational methods and measures taken at specific time points, to ensure adequate learning of the technique, is required in future studies.

This study has some limitations. For example, because the present study was conducted under a simulated scenario, different from clinical settings, the participants were not subject to time pressures or psychological factors; such as nervousness and anxiety that may be experienced in severe emergency situations. As previous studies have reported that these factors reduce performance, the results from this study may have overestimated the performance of the operators (Borges et al., 2010; Takamura, Kikuchi, & Inaba, 1999).

Furthermore, because we evaluated the performance in a simulated situation using a photograph and manikin, the results may differ with human subjects. The anatomy in the photograph allows easy visualization of laryngeal structures, whereas the training manikins tend to have a CTM anatomy that is easy to discriminate by touch. This is confirmed by the high CTM identification rates in this study, which exceed (by a large margin) the rates in clinical series (Bair & Chima, 2015; Hiller et al., 2016). This shows that manikin CTM identification is not a substitute for anatomic assessment of humans. Therefore, investigations of the palpation method should be performed with the human neck, in order to obtain results that can be translated to clinical settings. There are various models used for training in emergency airway management. The tracheotomy trainer has been shown to be effective for training in basic techniques (Friedman, You‐Ten, Bould, & Naik, 2008), and it is a feasible and relatively inexpensive training model compared with training with pig trachea (Cho et al., 2008). In training procedures using cadavers, formalin treatment of the cadavers has been reported to distort the skin and other tissues, interfering with the palpation techniques used for identifying anatomical landmarks (Eisma, Mahendran, Majumdar, Smith, & Soames, 2011). Despite the limitations discussed, training using the neck photograph and tracheotomy trainer in this study is still important to provide basic anatomical knowledge to students, such as the positional relationship of the CTM. Accurate knowledge of anatomy, a clear understanding of the procedures performed in airway management methods such as intubation and ventilation, and good practical skills are necessary to implement emergency airway management promptly and effectively (Hamaekers & Henderson, 2011). We suggest that students be trained first using neck photographs, then manikins such as the tracheotomy trainer, and finally pig trachea and cadavers. Simulation education, with high fidelity, has been shown to influence decision‐making in the CICV scenario, greatly improving the time to start the emergency airway management and the time to accomplish oxygenation (Borges et al., 2010). Previous studies have suggested that the minimum number of cricothyrotomy trials required to achieve basic proficiency is 5 (Greif, Egger, Basciani, Lockey, & Vogt, 2010; Wong, Prabhu, Coloma, Imasogie, & Chung, 2003). Furthermore, one report recommended that training should be repeated once every 6 months to ensure sufficient skill levels (Kuduvalli, Jervis, Tighe, & Robin, 2008). Therefore, regular training covering each feature could be a countermeasure for airway obstruction.

Finally, the extent and time period of sustenance of correct CTM identification rate (after the lecture and practicum) are unknown, as we were only able to follow up 16 days after the lecture. We suggest that future studies should investigate this.

## 6 CONCLUSIONS

In conclusion, the rate of successful CTM identification with the neck photograph and tracheotomy trainer, in fifth grade dental students, was low before undergoing a practicum in anesthesiology. However, following the lecture and practicum in a small class, most of the students were able to identify the CTM successfully. This practicum and lecture clarified the importance of penetrating the midline during cricothyrotomy to the students, whereas the structure of the learning procedure enabled the students to properly understand the anatomical structures of the midline and highlighted the importance of repetitive training for successful CTM identification.

## FUNDING INFORMATION

No funding information provided.

## CONFLICTS OF INTEREST

None declared.
